# Assessment of Efficacy of BLIS-Producing Probiotic K12 for the Prevention of Group A Streptococcus Pharyngitis: a Short Communication

**DOI:** 10.1007/s12602-018-9398-7

**Published:** 2018-02-20

**Authors:** Francesco Di Pierro

**Affiliations:** Velleja Research, Viale Lunigiana 23, 20125 Milan, Italy

**Keywords:** Oral probiotic, Group A streptococci, Pharyngitis, Carriage, *Streptococcus salivarius* K12

## Abstract

The neutral outcome of the recently reported school-based trial of probiotic K12 (The effect of the oral probiotic Streptococcus salivarius K12 on group A streptococcus pharyngitis: a pragmatic trial in schools) can be attributed at least partially to several readily identifiable confounding factors. Mainly, the execution and outcome were negatively impacted by (a) the suboptimal efficacy and frequency of K12 administration, (b) the failure both clinically and microbiologically to adequately diagnose and distinguish active group A streptococci (GAS) pharyngitis from harmless GAS carriage, and (c) the exceptionally low occurrence of GAS in this population at the time of the probiotic intervention due to recent high-intensity antibiotic exposure.

The essentially neutral outcome of the recently-reported, New Zealand (NZ)-based, placebo-controlled study of “The effect of the oral probiotic *Streptococcus salivarius* K12 (K12) on group A streptococcus pharyngitis: a pragmatic trial in schools” [1] might, without more careful analysis, appear to be quite at variance with our own and other previously-published findings of a substantial benefit associated with the use of this probiotic by children having a recent history of increased *susceptibility* to streptococcal pharyngotonsillitis [2–5]. However, as shown in the recent past [6], I believe that several important factors contribute to the apparently different outcomes of these studies and that an awareness of these factors is especially important for researchers working within this now rapidly-evolving field of probiotic health-care intervention.

Whereas our own applications of probiotic K12 have indicated a reduction in the occurrence of clinically diagnosed pharyngitis [2–5], the NZ trial did not incorporate any formal clinical assessment of the children’s sore throats [1]. Rather, it relied on detection of group A streptococci (GAS) in throat cultures as evidence of their causal association with the child’s pharyngitis. However, the challenge with this method is to correctly correlate a positive throat culture with true clinical infection [7]. There are of course many bacterial and viral causes of pharyngitis other than GAS [8]. Indeed, GAS most commonly live in the throat in a harmless carriage state, especially in young schoolchildren [9]. In the NZ trial, no distinction was reported between throat cultures having small (probable carriage state) numbers of GAS and those having large, infection-associated levels of GAS [1].

Furthermore, the subjects in this trial had also recently been part of another study in which intensive antibiotic intervention had been used to reduce GAS levels [10]. One outcome of this was that the occurrence of GAS in the children’s throat cultures was at a very low level (6.6%) throughout the probiotic trial. By comparison, a previous survey of NZ schoolchildren in another region characterised by a low occurrence of GAS sequelae showed that 24% of pharyngitis-negative children carried throat culture-detectable GAS [11]. The concern is that some (possibly most?) of the GAS detected in this present study represent asymptomatic carriage rather than acute infection. Since *S. pyogenes* carriers do not have evidence of disease or of an immune response due to *S. pyogenes*, they are not believed to be at risk for non-suppurative complications such as rheumatic fever or acute glomerulonephritis [12]. It is important to note that the lantibiotic-mediated competitor killing activity of *S. salivarius* K12 is more effective against GAS cells that are relatively rapidly multiplying, as are those actively causing a throat infection, and not against GAS cells that are present in a harmless carriage state, functionally indistinguishable from other members of the host’s indigenous oral microbiota [13].

It is important to note also that the subjects in this school-based study did not dose with BLIS K12 on weekends or in school holidays [1], resulting in an overall dosing adherence rate of only 38%; this value is very far from the ones (> 95%) shown in our studies [2–5]. Moreover, as shown also in studies aimed to *demonstrate* the action of K12 in adults [14] or in otitis media prevention [15]; the recommended dosing schedule for probiotic *S. salivarius* K12 is for it to be taken daily just prior to sleep, since the reduced saliva flow at night will then favour colonisation. In this study, the administration of K12 was incorporated within a busy classroom setting, undoubtedly making successful colonisation much more challenging.

Finally, to get a good colonisation rate with the oral probiotic K12, children have to be properly instructed to let the K12-tablet dissolve slowly in the mouth and have to be also carefully instructed not to chew the tablets or to swallow them whole. Furthermore, they should not drink or swallow anything else just following the use of the product. These procedures are quite difficult to be respected in very young children, while are likely easier to understand in older ones. Indeed, in the NZ trial, children older than 10 have had anyway a protection rate from GAS > 30% in comparison to a protection of about 15% in children between 7 and 9 and no protection at all in children below 7 [1].

In summary, the essentially neutral outcome of the recently reported school-based trial of probiotic K12 can be attributed at least partially to several readily identifiable confounding factors. Mainly, the execution and outcome were negatively impacted by (a) the suboptimal efficacy and frequency of K12 administration, (b) the failure both clinically and microbiologically to adequately diagnose and distinguish active GAS pharyngitis from harmless GAS carriage, and (c) the exceptionally low occurrence of GAS in this population at the time of the probiotic intervention due to recent high intensity antibiotic exposure. An additional factor that could possibly have influenced the outcome of this study concerns the authors’ disclosure that small numbers of contaminating K12 cells were found also to be present in the placebo tablets [1].

To better maximise the role exerted by the oral probiotic K12 in controlling *Streptococcus pyogenes* pharyngo-tonsillar clinical recurrences one should take into account fundamentally the following points:

1. Storing the strain between 2 and 8 °C;
2. Administering the strain during night hyposalivation;
3. Favouring slow dissolution of the strain-releasing tablet;
4. Not chewing the tablet;
5. Not swallowing the tablet;
6. Not drinking or eating anything after using the product;
7. Administering the product continuously for at least 90 days (longer is better);
8. Not treating carriage states;
9. Not co-administering K12 along with antibiotics (K12 is antibiotic sensitive) [16];
10. If possible, administering the probiotic strain as soon as antibiotic treatment is finished to improve the chances to get an immediate and better colonisation [6].

In conclusion, what the study has surely demonstrated is that intermittent classroom-based administration of K12 has not significantly impacted on the low numbers of asymptomatically carried GAS. However, the body of evidence for the efficacy of probiotic K12 in prevention of GAS pharyngitis remains overwhelmingly positive [2–5, 14, 15].
